# The nutritional support to prevent sarcopenia in the elderly

**DOI:** 10.3389/fnut.2024.1379814

**Published:** 2024-05-09

**Authors:** Attilio Giacosa, Gaetan Claude Barrile, Francesca Mansueto, Mariangela Rondanelli

**Affiliations:** ^1^CDI (Centro Diagnostico Italiano), Milan, Italy; ^2^Department of Public Health, Experimental and Forensic Medicine, University of Pavia, Pavia, Italy

**Keywords:** elderly sarcopenia, leucine, muscle mass, muscle protein synthesis, muscle strength, omega-3 fatty acids, vitamin D, whey protein

## Abstract

Sarcopenia has been described as a muscle disease, with multiple adverse consequences on human health. Recommendations aimed at supporting awareness, prevention, early detection and treatment of this disease are needed. This review focuses on the epidemiology, pathophysiology and early detection of elderly sarcopenia. As far as treatment is concerned, physical activity and nutritional support are specifically evaluated. An individually tailored resistance exercise training program appears to be crucial for a positive outcome of the sarcopenia prevention and treatment. The nutritional intervention is mostly based on the supplementation with high-quality proteins (i.e., whey protein) in order to increase the intake of essential amino acids and in particular of leucine. In addition, of relevant importance appears to be the supplementation with vitamin D, with omega-3 fatty acids and probiotics. This review evaluates the results of the most qualified studies on the nutritional supplementation of sarcopenic elderly subjects and shows that promising results have been achieved in community elderly subjects, or subjects followed in rehabilitation centers and in nursing homes, with additional resistance exercise programs.

## 1 Introduction

Sarcopenia is a muscle disease, (1) characterized by a progressive and generalized alteration of skeletal muscles, with a reduction in muscle mass and muscle strength. Sarcopenia is associated with physical disability, poor quality of life and increased mortality (2).

The prevalence of sarcopenia is high in the elderly population of both sexes. The epidemiological data show a high percentage of sarcopenia either among aging communities (5–10%) (3–5) or among elderly residents living in care homes (15–30%) (3, 5), or hospitalized in acute care wards (37%) (3, 6). For elderly patients followed in a rehabilitation setting the rate of sarcopenia goes up to 76% (7). Additionally, sarcopenia may coexist with obesity in the form of sarcopenic obesity, due to the frequent sedentary lifestyle in adult and elderly population (8). Sarcopenia is tightly related to aging, malnutrition, sedentary lifestyle, low physical activity and chronic diseases (9). It is associated with negative physical conditions because it favors clinical frailty (10, 11) and it increases falls (12) mortality, disability and institutionalization (13–15).

**Figure 1:**
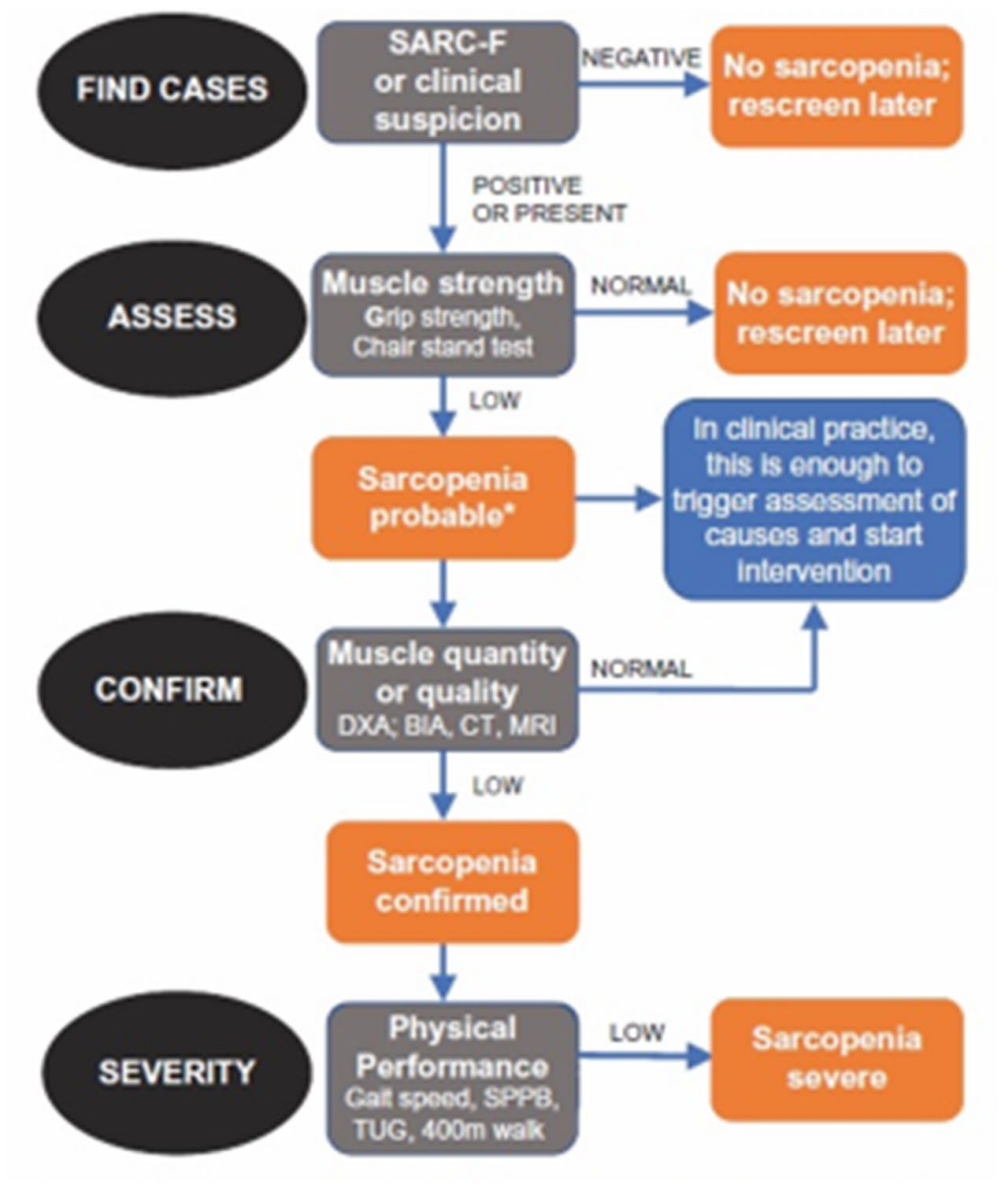
Diagnostic algorithm of sarcopenia produced by the European Working Group on Sarcopenia in Older People (7).

Although the negative effects of sarcopenia on human health and on social and healthcare costs have been widely demonstrated (16–19), this clinical problem is often under considered and undertreated, (7) (Figure 1).

Many recent studies evaluated the nutritional approach to treat sarcopenia in the elderly (20–23).

The objective of this review is to focus on a recommended muscle-targeted intervention—namely a whey protein, leucine, vitamin-D, Omega-3-fatty acid and probiotic supplementation—for the prevention or treatment of sarcopenia (9, 24, 25).

## 2 Pathophysiology of sarcopenia

Aging is physiologically associated with a decrease in muscle mass that represents a physiological process. This reduction is roughly 8% every ten years after the age of 40 and 15% after 70 years (3). The increase of inflammation and of anabolic resistance as well as a reduction of the protein intake and of physical activity are the main causes of this pathophysiological event (9, 24, 26, 27). The increased amino acids (AAs) splanchnic extraction represents an additional reason of their reduced availability in aging (25). In addition, after short term hospitalization or bed rest, the synthesis of muscle proteins is reduced by 30% in elderly subjects, with a loss of 1 kg of muscle mass in 3 days; whereas, after four weeks in similar conditions, young adults show a muscle loss of 0.5 kg (28).

Various new substances that could improve the muscle anabolism with modulation of androgen receptors or of the myostatin–activin process are being studied (27). Myostatin and activin are negative regulators of muscle growth and are now used as markers of loss of muscle mass, that is of sarcopenia (29). In any case, it must be emphasized that the adequate nutritional intake is the fundamental requirement to ensure the optimization of the muscle mass and of its function in the elderly. The scientific evidence available today indicates that an appropriate intake of proteins, an adequate physical exercise and vitamin D supplementation when it is deficient, are the fundamental nutritional requirements to achieve this goal (9, 24, 25). For people older than 65 years it is recommended a daily intake of 1–1.2 g/kg of proteins every day, with a higher amount of proteins (1.2–1.5 g/kg/day) when an inflammatory pathology is present. Proteins should mostly be of high quality, such as whey proteins, with high content of essential amino acids (EAAs) and rich in leucine (24, 25). At each meal, the intake of proteins of high quality should vary from 25 to 30 grams, with a leucine intake ranging from 2.8 to 3 grams and this should be provided at least twice a day (24, 30, 31). The minimum daily intake of leucine should be of 78.5 mg/kg (24, 30, 31). The high presence of EAAs and the fast digestion process of whey protein ensure that this protein source guarantees a marked anabolic efficacy (32) leucine has proven to be particularly effective in favorably modulating protein turnover and anabolism (30, 33).

Specific foods for special medical purposes (FSMP) should be provided when the food intake is inadequate. Various studies showed that oral supplementation with whey protein rich FSMP is followed by the greatest post prandial concentration of plasma AAs and by the greatest muscle protein synthesis, when compared with any other protein source (34, 35). This effect is demonstrated either with additional supplementation of leucine or not, either with additional increase of the energy supply or not and either with additional physical exercise or not (34, 35).

Beta-hydroxy beta-methylbutyrate (HMB), is a metabolite of leucine and it has been shown to be useful in the attenuation of the muscular mass loss, muscle strength and muscle function (36). The pharmacokinetics of leucine are characterized by a fast absorption and distribution, while HMB shows a slow metabolism with longer promotion of muscle protein synthesis (MPS) and lower breakdown rates (36).

The supplementation with essential amino acids also appears of great interest since these amino acids are rapidly assimilated by the digestive system without consumption of energy, that is of adenosine triphosphate (ATP), according to the blood /cell cytoplasm gradient. The more rapid the increase in concentration in the blood, the faster EAAS enter the cell. Finally, the intake of essential amino acids in free form is a more efficient anabolic stimulus than the intake of an equal quantity in the form of proteins (37).

Vitamin D has been demonstrated to be effective in various ways on muscle recovery and MPS (38) and its synergic effect with leucine in stimulating protein anabolism has been well described (39). Therefore, by virtue of the frequent finding of vitamin D deficiency in elderly subjects, a supplementation of at least 800–1,000 IU every day of vitamin D should be provided in sarcopenic elderly subjects (40–42). Also, omega-3 fatty acids can reduce muscle loss and favor muscle synthesis (43) on muscle mass has been shown in various animal models and *in vitro* (44–46) as well as in human experiments, regardless of the anti-inflammatory action (47).

Recently, a gut-muscle correlation has been described (48). Gut microbiota could mediate the correlation between nutrition and aging by regulating the host’s immune function, metabolism, insulin activity and gene expression (49, 50), and gut microbiota has been correlated with physical performance of elderly subjects (51, 52). Various studies have shown a correlation between gut microbiota composition and physical performance in the older population (51, 52). Aged rats supplemented with *Lactobacillus paracasei* PS23 showed a reduced muscle loss (53) and reduced cognitive decline (54). Therefore, a probiotic supplementation could be useful to favorably influence gut microbiota in elderly subjects with sarcopenia.

## 3 Efficacy trials

Among the numerous studies on the efficacy of FSMP useful for muscular mass synthesis, the most qualified clinical trials, with randomized and controlled study protocols and high number of recruited patients, are here reported.

In the PROVIDE study, malnourished older patients with sarcopenia living independently were randomized to vitamin D and leucine-enriched whey protein nutritional supplement or to an isocaloric control product, given twice daily for 13 weeks. Although the trial did not reach a significant between group difference in SPPB (Short Performance Physical Battery) and grip strength, the chair stand test as well as the appendicular muscle mass showed a significant improvement in patients supplemented with the muscle targeted food for special medical purposes (MT-FSMP) (55).

In the randomized study performed by Chanet et al. to evaluate the effect of a standardized breakfast supplemented with vitamin D and leucine enriched whey protein or a non-caloric placebo in healthy older men, a significant benefit towards appendicular lean mass was observed only in the test group at the end of a 6 week intervention (56).

In another controlled study, 130 sarcopenic elderly subjects were randomly supplemented with one serving per day containing 22 grams of whey protein, 10.9 grams of essential amino acids (including 4 grams of leucine), 100 IU of vitamin D or an isocaloric quantity of maltodextrin for 90 days. A personalized program of moderate-intensity physical activity was planned at the same time for all subjects. The intervention group showed a significantly higher increase in muscle mass, handgrip strength and physical performance, when compared with the control group (57). In addition, the intervention group showed lower CRP values and improved quality of life scores.

The IRIS randomized study evaluated the efficacy of a whey protein-based nutritional formula, enriched with leucine and vitamin D, twice daily, in addition to a standard hospital diet and a physical exercise rehabilitation program, in older in-patients with sarcopenia and compared it with an isocaloric control formula supplemented for 8 weeks (57). The four meters gait speed as well as the chair stand test, the TUG (timed up and go test), the SPPB (short physical performance battery), the Barthel index, the handgrip strength, the ADL (activity daily living), the QoL (quality of life) and appendicular muscle mass were significantly improved only in the intervention group. Moreover, CRP levels, healthcare resource consumption, and length of stay in hospital were lowered only in the intervention group (58).

Sarcopenia associated with obesity (8) is an interesting topic because weight loss is beneficial in overweight older subjects, but it may be associated with loss of skeletal muscle mass and sarcopenia. Verreijen et al. showed that a 13 week weight loss program was followed by a similar weight loss and fat mass loss in older obese subjects supplemented with whey protein, leucine and vitamin D as compared to the control group; but the intervention group showed an increase in appendicular muscle mass, whereas the control group evidenced a decrease in the same variable (59).

The effect on muscle mass of a two-month randomized intervention in sarcopenic elderly subjects with a MT-FSMP composed of 500 mg of omega-3 fatty acids, 2.5 g of leucine and probiotic *Lactobacillus paracasei* PS23 (30 billion, freeze dried), versus a placebo control group, has been evaluated by Rondanelli et al. (60). The obtained data showed a significant increase of appendicular lean mass, of the Tinetti scale, of the SPPB total score and of the handgrip strength in the intervention group, while the control group did not show any difference. Moreover, the comparison between the two studied groups demonstrated a significant decrease of the visceral adipose tissue and a significant increase of valine, leucine, isoleucine, and total amino acids only in the test group (60).

## 4 Discussion and conclusion

Several studies have been conducted with different muscle targeted foods for special medical purposes in older subjects with sarcopenia. The obtained results demonstrate that the intervention with these products, possibly in combination with a physical exercise program, promotes muscle protein synthesis and promotes the increase of muscle mass and muscle strength, and improves the physical performance of elderly sarcopenic subjects (Table 1). In addition, multiple studies show that these interventions prevent the muscle mass loss in subjects at high risk of becoming sarcopenic (Table 1).

**Table 1:** Nutritional and physical interventions to treat or prevent elderly sarcopenia.

Critical Factor	Intervention	References
Low protein intake	Increased protein intake (1–1.5 g/Kg/day) “Fast” proteins (Whey protein): 25 g each meal at least twice a day	(24, 25)
Low muscular protein synthesis	Increased leucine supply 2.5–2.8 g/meal at least twice a day	(34, 35)
Low serum vitamin D	Increased vitamin D ≥800 IU/day	(38–42)
Low intake of omega-3 fatty acids	Increased omega 3 fatty acids ≥500 mg/day	(43–46, 61–63)
Microbiota unbalance	Probiotic supplementation *Lactobacillus paracasei* PS23	(51–53, 60)
Sedentarism	Increased physical activity ≥30 min/day	(24, 25)

The efficacy of MT-FSMP is higher in association with physical activity and in particular with resistance exercise programs (57–59, 64, 65) have also been shown in subjects who do not increase physical activity (65–67). This is a great advantage for individuals who, for various reasons, have difficulty in carrying out rehabilitative physical activity programs. The same muscular benefits have been demonstrated in elderly obese individuals who need to implement nutritional measures aimed at reducing body weight (59, 68, 69). The efficacy of MT-FSMP has been shown in heterogeneous patient populations, (70, 71) and these nutritional interventions showed to reduce healthcare resource consumption in rehabilitation (58). To detect the recovery of muscle mass, the minimum duration treatment should be 4–8 weeks. Future research should evaluate the efficacy of long-term supplementation. Data on its tolerability up to 6 months have been provided (65, 72, 73). Moreover, this nutritional intervention could prevent the oxidation of dietary proteins as a source of energy (74). The effect of combined, multifactorial interventions (MT-FSMP and physical activity) would be desirable due to their synergistic effects. It has to be stated that, according to the triage theory of Bruce Ames, it could be advisable to supplement all the population with clinical signs of sarcopenia (75).

In conclusion, available data indicate that a muscle-targeted oral nutritional supplementation constitutes an effective treatment of sarcopenia and should be offered as a first-line therapeutic intervention in these subjects. The positive outcome of the nutritional intervention may be additionally increased when a targeted resistance exercise program is added. This intervention appears useful also to prevent sarcopenia in high-risk elderly subjects.

## Author contributions

AG: Conceptualization, Writing – original draft. GB: Writing – review & editing. FM: Writing – review & editing. MR: Writing – review & editing.
